# Aortic Isthmus Doppler Velocimetry in Fetuses with Intrauterine Growth Restriction: A Literature Review

**DOI:** 10.1055/s-0040-1710301

**Published:** 2020-05

**Authors:** Mariana Martins Ferraz, Flávia do Vale Araújo, Paulo Roberto Nassar de Carvalho, Renato Augusto Moreira de Sá

**Affiliations:** 1 Fetal Medicine Post Graduation, Universidade Estácio de Sá, Rio de Janeiro, RJ, Brazil; 2 Fetal Medicine Department, Instituto Fernandes Figueira, Rio de Janeiro, RJ, Brazil; 3 Clínica Perinatal, Rio de Janeiro, RJ, Brazil

**Keywords:** doppler velocimetry, doppler, aortic isthmus, intrauterine growth restriction, intrauterine growth retardation, placental insufficiency, dopplervelocimetria, doppler, istmo aórtico, crescimento intrauterino restrito, crescimento intrauterino retardo, insuficiência placentária

## Abstract

Intrauterine growth restriction (IUGR) is associated with poor perinatal prognosis and a higher risk of stillbirth, neonatal death, and cerebral palsy. Its detection and the evaluation of its severity by new Doppler velocimetric parameters, such as aortic isthmus (AoI), are of great relevance for obstetrical practice. The AoI is a vascular segment that represents a point of communication between the right and left fetal circulations. It is considered to be a functional arterial shunt that reflects the relationship between the systemic and cerebral impedances, and has recently been proposed as a tool to detect the status of hemodynamic balance and prognosis of IUGR in fetuses. In the present review, we noticed that in healthy fetuses, the AoI net flow is always antegrade, but in fetuses with IUGR the deterioration of placental function leads to progressive reduction in its flow until it becomes mostly retrograde; this point is associated with a drastic reduction in oxygen delivery to the brain. The more impaired the AoI flow is, the greater is the risk of impairment in the Doppler velocimetry of other vessels; and the alterations of the AoI Doppler seem to precede other indicators of severe hypoxemia. Although there seems to be an association between the presence of retrograde flow in the AoI and the risk of long-term neurologic disability, its role in the prediction of perinatal morbi-mortality remains unclear. The AoI Doppler seems to be a promising tool in the management of fetuses with IUGR, but more studies are needed to investigate its employment in clinical practice.

## Resumo

O crescimento intrauterino restrito (CIUR) está associado a um prognóstico perinatal adverso, com maior risco de óbito intrauterino e neonatal, bem como de paralisia cerebral. Assim, sua detecção e a determinação de sua gravidade por novos parâmetros Dopplervelocimétricos, como o istmo aórtico (IAo), são de fundamental importância na prática obstétrica. O IAo é um segmento vascular que representa um ponto de comunicação entre os sistemas circulatórios fetais esquerdo e direito. É considerado um *shunt* arterial funcional, capaz de refletir a relação entre as impedâncias dos circuitos cerebral e sistêmico, e foi proposto como uma ferramenta para detecção do status do equilíbrio hemodinâmico e do prognóstico de fetos com CIUR. Na presente revisão, observou-se que, em fetos saudáveis, o fluxo predominante no IAo é sempre anterógrado; mas em fetos com CIUR a deterioração do estágio de insuficiência placentária acarreta reduções progressivas no fluxo ístmico até este apresentar sentido predominantemente retrógrado e levar a uma drástica redução no aporte de oxigênio ao sistema nervoso central. Quanto mais alterado estiver o fluxo no IAo, maior a chance de haver alteração na Dopplervelocimetria de outros vasos; e as alterações no Doppler do IAo parecem preceder outros indicadores de hipoxemia severa. Embora o fluxo retrógrado no IAo pareça se correlacionar com maior risco de alteração no desenvolvimento neurológico a longo prazo, ainda não está claro o seu papel na predição de morbimortalidade perinatal. O Doppler do IAo parece ser um parâmetro promissor no manejo do CIUR; entretanto, mais estudos são necessários para avaliar seu emprego na prática clínica.

## Introduction

Intrauterine growth restriction (IUGR) can be defined as the failure of a fetus to achieve its full growth potential and can be caused by placental, chromosomal, or environmental factors. This condition is associated with a poor perinatal outcome, and it is responsible for 50% of intrauterine demise; it elevates the risks of intrapartum fetal distress, emergency caesarian section, cerebral palsy, and perinatal death.^1^ ^2^ Therefore, its detection and the determination of its severity are of major relevance for obstetric practice and constitute an opportunity for intervention to prevent unpleasant perinatal outcomes.

Placental insufficiency is the main etiological factor responsible for IUGR, affecting up to 15% of all pregnancies.^3^ Such insufficiency provokes significant changes in fetal circulation that induce mechanisms of hemodynamic compensation.^2^ ^3^ ^4^ Comprehension of this adaptive process of vascular redistribution is the key to the appropriate management of IUGR and explains the crucial role of Doppler velocimetry in the study of gestational vessels. Since there is no effective intrauterine treatment for IUGR caused by placental insufficiency, the aim of Doppler fetal monitoring is to detect the moment of hemodynamic decompensation at which the risk of maintaining the concept of a hypoxic environment should be balanced against risks related to prematurity caused by the interruption of gestation.^2^ ^3^ ^5^ ^6^

While insonation of maternal vessels, such as the uterine arteries and some fetal vessels—like the umbilical artery, middle cerebral artery, and ductus venosus—is well established in clinical practice, other vessels have recently been proposed to be useful in the prognostic evaluation and decision-making process of selecting the ideal moment for delivery.^2^ ^4^ ^6^ ^7^ One such case is the aortic isthmus (AoI), which was first described in 1993 and was proposed as a new tool to monitor hemodynamic and metabolic balance in fetuses with IUGR.^8^

The AoI is a vascular segment located between the origin of the left subclavian artery and the connection of the ductus arteriosus in the descendent aorta; it represents a point of communication between the right and left fetal circulations. Because of this anatomical characteristic, the AoI is considered to be a functional arterial shunt, reflecting the relationship between impedances of the cerebral circuit (supplied by the left ventricle) and the systemic circuit (perfused by the right ventricle). Changes in AoI blood flow could arise as a result of an imbalance between the vascular adaptations of these two parallel circulations. A hemodynamic impairment capable of overwhelming the systemic resistance to the detriment of the central impedance could reverse the direction of flow in the AoI, leading to poorly oxygenated blood being ejected from the right ventricle and to the central nervous system, thus causing cerebral hypoxia. Considering this singularity, the AoI Doppler has been the focus of many investigations and has been highlighted as a possible marker of the moment of critical hemodynamic decompensation that gives rise to cerebral hypoxia, especially in fetuses with late IUGR.

The aim of this study was to review and discuss our current knowledge relating to Doppler velocimetry in the AoI along the time course of pregnancy in fetuses with IUGR.

## Review Results

This is a review of the literature retrieved via searches of the MEDLINE/PubMed, and the LILACS and Scielo databases for articles containing the following keywords: *aortic isthmus* [AND] *intrauterine* *growth* *restriction* [OR] *intrauterine* *growth* *retardation*.

Because this review relates to a recent topic, no restriction criteria were used in regard to the year of publication; the earliest article was published in 1993 and the latest was published in 2018. No language filter was applied, and the articles found and included in this study were published in either English or French.

In total, 46 articles were identified by the initial search. Of these, 11 were excluded because they were considered to be irrelevant to the main theme, and another 10 were excluded because they were review/opinion papers or because they showed evidence of methodological problems. We searched the bibliographies of the 25 remaining studies for citations of interest, which led to the inclusion of 6 additional articles. Consequently, this review is based upon 31 published articles.

## Discussion

### Aortic Isthmus Doppler in Normal Fetuses

The study of the aortic isthmus (AoI) began with animal experiments in which surgical models of placental resistance were established using fetal lambs. This model allowed the analysis of flow parameters in the AoI and the umbilical artery (UA) at different levels of placental insufficiency.^8^ This experiment showed that progressive mechanical compression of the umbilical vein (thus promoting resistance to placental flow) provoked changes in the volume and direction of the diastolic flow, which began earlier and were more pronounced in the AoI than in the UA. This association highlighted the AoI as a sensitive marker of the status of umbilical circulation.^8^

Afterwards, the same group repeated a similar experimental protocol and correlated the changes in AoI flow with the carotid oxygen delivery to the central nervous system.^9^ The data showed that while the net blood flow in the AoI was still antegrade, the content of oxygen in the carotid artery remained relatively constant. However, when the net flow became retrograde, there was an abrupt decrease in the level of carotid oxygenation, thus indicating the moment of decompensation when cerebral hypoxia was induced.

**Fig. 1:**
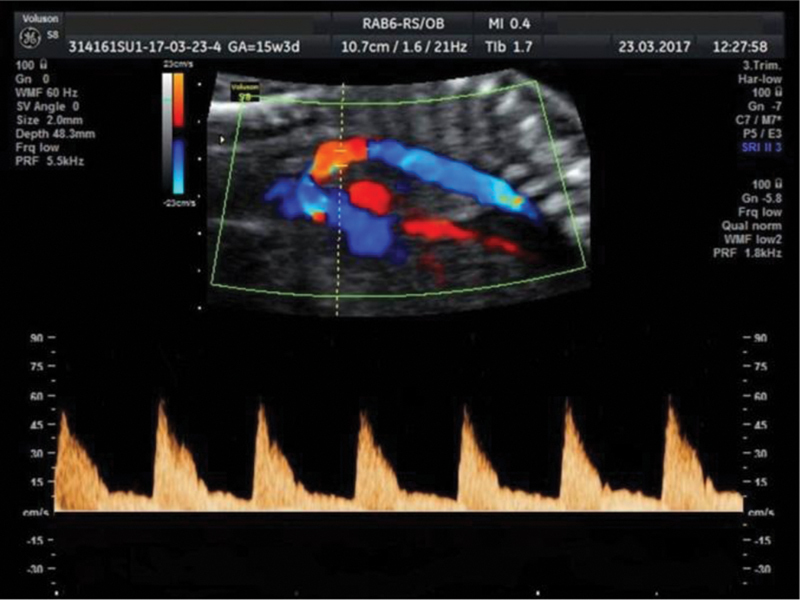
Aortic isthmus Doppler of a 15-week fetus showing a smooth and uniform deceleration without incisura.

**Fig. 2:**
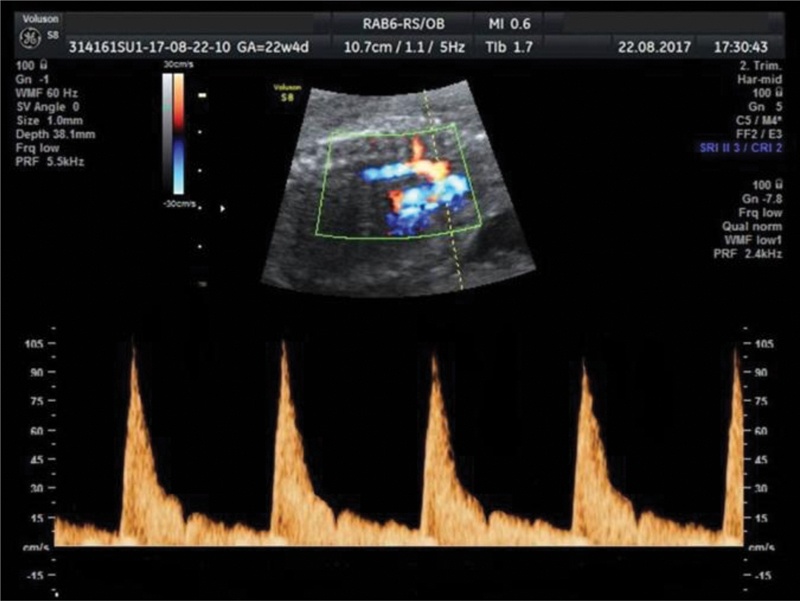
Aortic isthmus flow profile of a 22-week fetus demonstrating an incisura formed by a sharp deceleration at the end of systole, followed by an acceleration during early diastole.

**Fig. 3:**
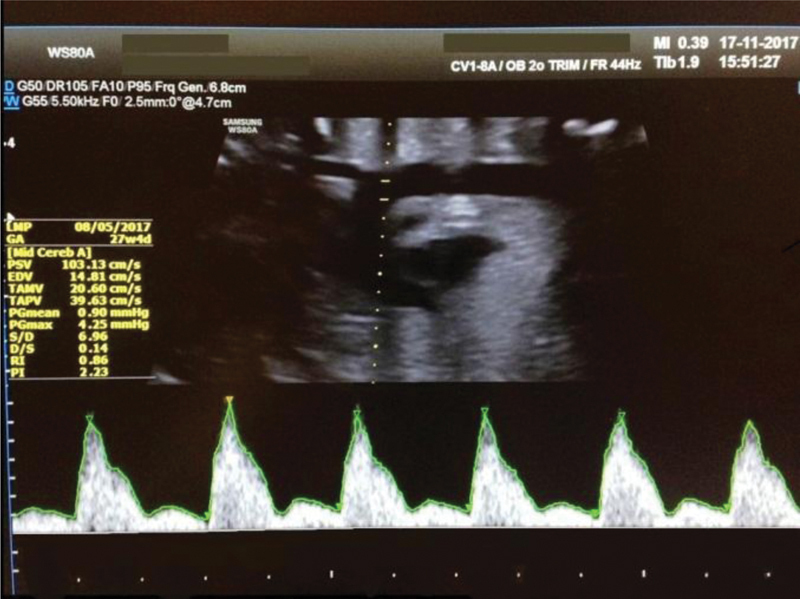
Aortic isthmus flow velocity waveform of a 27-week fetus with a more pronounced incisura.

**Fig. 4:**
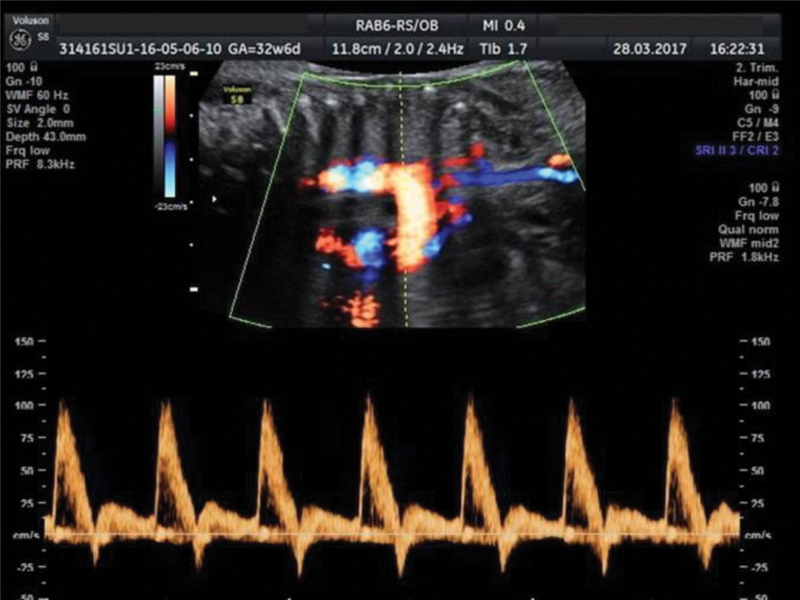
Aortic isthmus Doppler of a 32-week fetus revealing a brief reverse flow at the beginning of diastole.

Following these promising initial results of animal experiments, the use of Doppler velocimetry in the analysis of the AoI became a subject of interest to researchers working on human fetuses. Observational studies were conducted with the aim of describing the characteristics of the AoI flow profile throughout gestation in fetuses without any pathology. It was shown that the flow velocity waveform in the AoI changes as pregnancy advances.^10^ After a typical quick systolic peak, observed at all gestational ages, the morphology of the wave deceleration, which marks the transition from systole to diastole, undergoes slight modifications. Before 20 weeks, this deceleration occurs in a smooth and uniform manner without the formation of incisura (Fig. 1). Between 20 and 25 weeks, a sharp deceleration is observed at the end of systole, which is followed by an acceleration during early diastole, thus forming an incisura (Fig. 2). From 25 to 30 weeks, this incisura progressively increases, reaching its nadir around zero velocity at ∼ 30 weeks of gestation (Fig. 3). After 31 weeks, there is a brief reverse flow at the beginning of diastole, which is more pronounced as gestation progresses (Fig. 4). However, it is not clear if this reverse flow represents a technical artifact or a physiological effect caused by the increase in flow from the right ventricle, which is known to occur at the end of systole around this gestational age.^10^ ^11^

Quantitative physiological changes, as measured by AoI Doppler velocimetric indices, have been documented throughout gestation in a range of observational studies, which determined useful reference values for each gestational age.^11^ ^12^ These studies identified an increase in the pulsatility index (PI) and in the peak systolic velocity (PSV) with the advancement of pregnancy as well as a reduction in diastolic flow.^11^ ^12^ The modifications in the AoI and UA indices were shown to be independent variables.^11^ There have been no cases of absent or net reverse diastolic flow documented for normal pregnancies.^12^

**Fig. 5:**
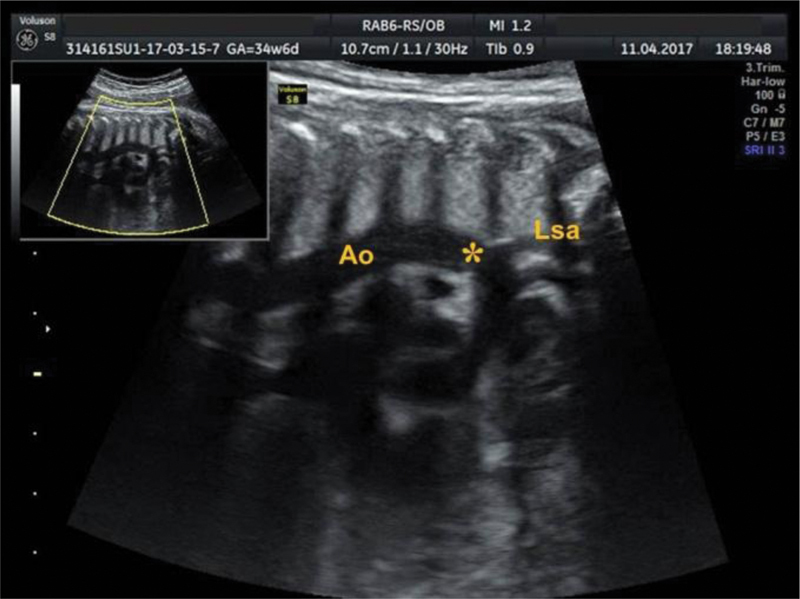
Sagittal plane of the Aortic isthmus; the gate (*) should be placed a few millimeters beyond the origin of the left subclavian artery (Lsa). Ao indicates Aorta.

**Fig. 6:**
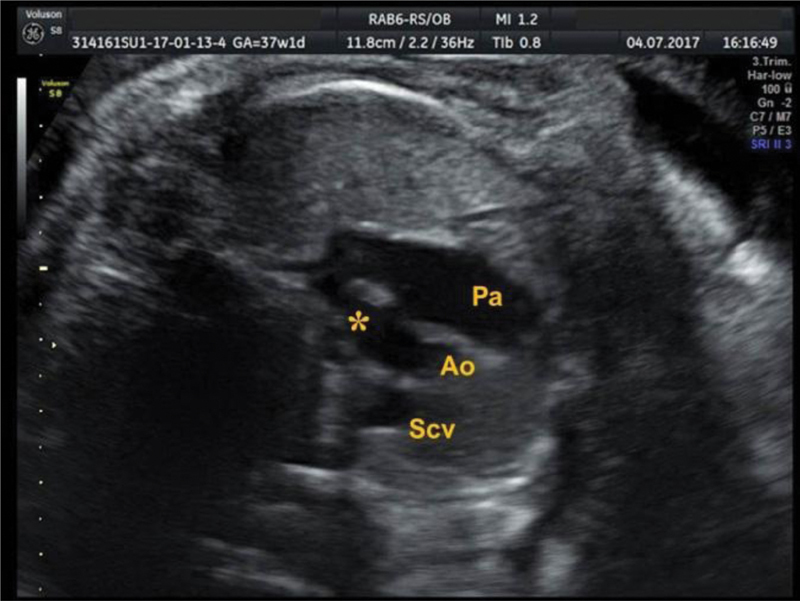
Transverse plane of the Aortic isthmus, in three-vessel view, with the gate (*) placed just before the edge of the V shape formed by the convergence of the ductus arteriosus with the aorta (Ao). Pa indicates pulmonary artery; and Scv, superior vena cava.

Another important issue in the investigation of AoI Doppler was the technical validation of this technique as a diagnostic method with good intra and interobserver reproducibility.^13^ ^14^ Both sagittal and transverse planes have proven to exert good reliability.^14^ ^15^ ^16^ The sagittal plane can be obtained with a 90° transducer rotation from the fetal heart 4-chamber horizontal view, thus providing a clear visualization of the aortic arch. The AoI flow can then be identified by placing the gate a few millimeters beyond the origin of the left subclavian artery (Fig. 5). The transverse plane can be evaluated from a viewpoint that includes the three vessels and the trachea and by identifying the convergence of the ductus arteriosus with the aorta; this forms a ‘V’ shaped image, and the gate should be placed just before the edge of the ‘V’ (Fig. 6).

Although the technical pattern of AoI insonation has been established, there has yet to be a consensus on the best index with which to measure its alterations. Ruskamp et al^12^ proposed the use of the isthmic flow index (IFI), calculated by the formula (S + D)/S, where S = systolic velocity integral and D = diastolic velocity integral. The IFI could then be classified as type I when its value is > 1, representing an antegrade flow in both systole and diastole; type II when the IFI is between 0 and 1, reflecting a diastolic reverse flow, but with a predominantly antegrade systolic flow; or type III when the IFI < 0, thus reflecting a predominantly retrograde flow.^12^ Even though this index is capable of highlighting changes in the direction of diastolic flow in a very sensitive manner, it involves a complex formula that requires manual calculation. Other authors have defended the application of the traditional PI, which is simpler and has proven sensitivity for detecting changes in AoI flow.^5^ ^11^ ^14^ ^15^ However, this index was developed to calculate flow impedance in a single artery and does not incorporate the complexity of the range of determinants that influence the AoI and cannot indicate the presence or quantify the retrograde component of AoI flow.^12^ ^13^ Some researchers, however, apply a simple qualitative analysis of wave flow; these researchers consider the presence of any diastolic flow as being normal and classify cases with an absent or negative diastole as being abnormal.^17^ Other researchers calculate the ratio of the systolic velocity integral to the retrograde diastolic velocity integral and define net antegrade flow when this ratio is > 1 and net retrograde flow when the ratio is < 1.^4^ ^7^ While quantitative indices allow the identification of more subtle and earlier changes, the qualitative evaluation of the flow direction allows the selection of more compromised fetuses with a greater risk of cerebral hypoxia.^14^

### Aortic Isthmus Doppler in Fetuses with Growth Restriction

Due to the diversity of methods used to measure the AoI flow, the comparison of results obtained by different studies represents a challenging task. However, it is still possible to draw some conclusions in regard to the compromised AoI Doppler data obtained from fetuses affected by IUGR.

Observational studies in fetuses with IUGR have shown that changes in AoI Doppler (described either qualitatively or quantitatively) were related to the degree of fetal hemodynamic compromise; the more altered the flow in the AoI was, the greater was the chance of changes occurring in other vessels.^5^ ^18^ ^19^ ^20^ By analyzing the Doppler velocimetry of 100 fetuses with IUGR and an UA-PI > p95, Sonesson and Fouron^18^ demonstrated that an absent/reverse diastolic flow was more frequently seen in the AoI than in the UA. Furthermore, when there was an absent/reverse diastolic flow in the UA, this effect was also present in the AoI but in a more pronounced manner. Benavides-Serralde et al^19^ observed that fetuses with an UA-PI < p95 maintained a normal IFI; however, progressive changes in UA flow were followed by greater changes in the AoI.

Other studies have compared AoI Doppler velocimetry in fetuses with IUGR with that of normal fetuses and have shown lower velocity indices (e.g., end diastolic velocity – EDV; peak systolic velocity – PSV; time-averaged maximum velocity – TAMXV) and higher resistance indices (e.g., pulsatility index – PI; resistance index – RI) in the fetuses with IUGR.^5^ ^21^

In regard to these findings, Cruz-Martinez et al^22^ attempted to investigate whether AoI Doppler could play a role in distinguishing between late IUGR fetuses and constitutionally small fetuses. These authors compared the Doppler velocimetry results of 178 small for gestational age (SGA) fetuses (with an estimated weight < p10 and an UA-PI < p95) with those of 178 fetuses with an adequate for gestational age (AGA) weight. They identified a significantly higher AoI-PI (3.84 vs 2.87; *p* < 0.01) in SGA fetuses, concomitant with a greater incidence of the indices above p95 (14.6% vs 5.1%). Of the fetuses with AoI net retrograde flow, none were classified as having AGA weight, 15.4% had neonatal acidosis, and 84.6% required emergency Caesarian section for fetal distress. This study found that a proportion of SGA fetuses showed AoI Doppler abnormalities that were associated with a poorer fetal prognosis, suggesting that AoI impairment could identify a group of fetuses at greater risk of adverse outcomes presumably attributed to late IUGR. However, a similar study comparing 72 AGA, 48 SGA and 10 IUGR fetuses did not identify any significant differences in terms of Ao-PI when compared across the three groups.^14^ It is likely that this discrepancy is due to the small number of patients in the second study; nevertheless, at the present time, it is not possible to consider AoI as a valid parameter to distinguish between constitutionally small fetuses and those with true growth restriction.

Some other studies have tried to longitudinally analyze the moment at which AoI Doppler changes are established in fetuses with IUGR and to determine its relationship with the onset of abnormalities in other vessels. Studies showed that an elevation of UA-PI precedes changes in the AoI by ∼ 11 to 20 days, but in the MCA-PI, this elevation occurs 7 to 15 days prior.^6^ ^23^ Alterations in the DV-PI succeed deterioration in the AoI by 2 to 7 days.^5^ ^6^ ^23^ ^24^ In observing a population of 31 fetuses with IUGR and UA absent/reverse diastolic flow, Rizzo et al^17^ demonstrated that in all cases involving DV reverse diastolic flow, there was also evidence of AoI retrograde net flow. Furthermore, of the fetuses with a DV-positive diastolic flow, those with AoI retrograde net flow were delivered earlier.

Despite the fact that several studies have investigated AoI Doppler changes in fetuses with IUGR and have arrived at similar conclusions, all of the studies were performed with a small number of patients. Only one previous study recruited a larger population and showed that a small impairment of the AoI did not appear to precede DV changes.^25^ This study formed part of a multicenter clinical trial that prospectively analyzed Doppler changes in 1,116 fetuses with gestational ages between 24 and 37 weeks, with an estimated weight < p10 but above 500 g. The detected prevalence of altered Doppler velocimetry was 46% in the UA, 27% in the MCA, 11% in the DV, and only 5% in the AoI. The low prevalence of AoI Doppler changes placed significant doubt on the potential role of this manifestation in clinical practice.

### Perinatal Outcomes

The physiopathology involved in the change of AoI flow direction appears to be related not only to the augmentation of peripheral vascular resistance and cerebral vasodilation but also to the failure of adaptive mechanisms underlying the redistribution of pulmonary blood flow to the systemic circulation.^26^ During an investigation of hemodynamic changes in fetuses with IUGR, Makikallio et al^4^ ^27^ compared the changes occurring in different fetal vessels that maintained an antegrade AoI flow with vessels showing a retrograde AoI flow. The data showed that placental function was equally affected, that the umbilical-cerebral ratio was similar between the two cohorts, and that the umbilical vein oxygen tension was equivalent.^4^ Moreover, the group showing antegrade AoI flow was able to redirect the blood ejected from the right ventricle by suppressing its supply to the pulmonary arteries and elevating the proportion of flow passing through the ductus arteriosus to the systemic circulation. Furthermore, the volume of blood directed from the right to the left atrium, through the foramen ovale, was greater in fetuses with antegrade AoI flow, allowing for a higher delivery of well-oxygenated blood to the left ventricle.^27^ In contrast, fetuses with retrograde AoI flow were not able to undergo these adaptations and maintained a flow distribution similar to fetuses that were not suffering from placental insufficiency, resulting in a lesser capacity for cerebral oxygenation.^4^ ^27^

These findings led to the assumption that the presence of retrograde flow in the AoI could be an indicator of poor fetal prognosis, including a higher risk of cerebral hypoxia and perinatal morbi-mortality. We identified 6 studies evaluating the value of AoI in predicting perinatal mortality; only one failed to show a statistically significant correlation.^28^ The other five studies reported a positive association between the risk of perinatal death and AoI Doppler change when this parameter was considered alone.^5^ ^23^ ^29^ ^30^ ^31^ However, using multivariate analysis and considering gestational age and the presence of Doppler changes in other vessels collectively, Hernandez-Andrade et al^30^ and Cruz-Lemini et al^31^ demonstrated that the power of AoI to predict mortality lost statistical significance. This was probably because alterations in the AoI occur relatively early in the sequence of vascular changes in response to hypoxic deterioration, preceding DV changes by approximately one week and exhibiting a poor capacity to predict late events such as mortality.^6^ ^30^ ^31^ Such instances require more acute markers of fetal acidosis, such as DV-PI.^30^ ^31^

Studies that attempted to correlate changes in the AoI with perinatal morbidity were even less conclusive, presenting significant heterogeneity in the parameters evaluated and conflicting results. Two studies collectively analyzed a range of unfavorable perinatal outcomes considered as total morbidity. One of these studies identified a positive association with the presence of AoI retrograde flow, while the other did not.^23^ ^28^ Three other studies investigated AoI as a predictor of the requirement of intensive neonatal care; these studies identified a consistently positive correlation.^5^ ^21^ ^29^ Of the three studies investigating UA-pH modification, none were able to identify a relationship with the AoI.^5^ ^23^ ^28^ Four studies considered the 5th minute Apgar score as a potential outcome, and only 2 successfully related retrograde flow in the AoI to a lower Apgar score.^5^ ^21^ ^23^ ^28^ One study found a greater risk of necrotizing enterocolitis, while two other studies did not.^23^ ^28^ ^32^ Consequently, AoI Doppler velocimetry has yet to demonstrate a clear and consistent role in the prediction of perinatal morbidity.

### Long-term Outcomes

Despite the lack of clarity in regard to the role of AoI Doppler velocimetry in predicting perinatal morbidity, this technique has been shown to be a good predictor of long-term neurological morbidity.^7^ ^33^ ^34^ Cruz-Martinez et al^33^ demonstrated a statistically significant association between the presence of AoI retrograde net flow and abnormalities found during intracranial neonatal ultrasound (both transient and late lesions). In another study, Fouron et al^7^ correlated AoI net retrograde flow with a higher risk of long-term neurodevelopmental deficit (relative risk = 2.05–confidence interval 95%; 1.49–2.83) and established a cutoff point to predict the risk of abnormal neurodevelopment. These authors proposed that an IFI value below 0.7 would be associated with a greater risk of impairment in late neurological tests, with a sensitivity of 55% and a specificity of 89%.^34^

## Conclusion

Since the publication of the first experimental studies in animals, there have been significant advances in the field of aortic isthmus Doppler velocimetry. The physiology of the AoI in normal pregnancies and the physiopathology in cases of placental insufficiency have been elucidated, and changes in the velocity flow profile in the AoI throughout gestation have been well described. It is clear that the predominant flow in the AoI is always antegrade in fetuses without any pathology and that as gestational age advances, there is a physiological reduction in its diastolic flow, resulting in a reduction in IFI and an elevation of AoI-PI. For fetuses affected by IUGR, there is a trend for the AoI to show lower velocity indices (e.g., PSV, EDV, and TAMX) and higher resistance indices (PI and RI). Furthermore, deterioration during the stage of placental insufficiency results in progressive reductions in AoI flow until this presents as a predominantly retrograde flow. At this point, there is a drastic reduction in the delivery of oxygen to the central nervous system. Studies have also shown that the more altered the AoI becomes, the greater is the chance of Doppler changes in other vessels and that the changes in AoI Doppler velocimetry appear to precede alterations that are indicative of severe hypoxemia, such as UA absent/reverse diastole and DV impairment. However, there are still many questions to be answered before this Doppler parameter can be endorsed by routine clinical practice. Despite validation of the AoI insonation technique, there is no uniformity in the method of flow measurement, thus compromising the comparability of results and the application of this technique across different centers. Although the presence of AoI retrograde flow appears to be related to a greater risk of long-term neurological impairment, its role in the prediction of perinatal morbi-mortality is not yet clear. Furthermore, there is insufficient data to support the application of this tool to distinguish between constitutionally small and late IUGR fetuses. It is also important to emphasize that the majority of studies performed thus far have involved a small number of patients and that the only previous study that examined a large population did not provide any evidence that AoI changes were of great importance. Finally, all of the studies included in this present review involved an observational design; as yet, there has been no clinical trial investigating the incorporation of AoI Doppler velocimetry in the obstetric decision-making process. No study was designed to compare AoI Doppler findings with abnormal computerized cardiotocography tracings and we cannot speculate if this tool could be replaced by AoI Doppler methods. AoI Doppler seems to be a promising tool in the guidance of the hemodynamic status of fetuses with IUGR, especially in cases where the acquisition of the Ductus Venosus is difficult, but the available evidence is still insufficient to support its incorporation in obstetrical protocols. Further studies are now needed to evaluate the application of the AoI in IUGR management and to investigate if this could play a role in predicting the best moment of delivery. Since there are no intrauterine treatments available for fetuses with growth restriction, it has become very important to establish the most opportune moment for delivery. This decision must balance the complications of prematurity against the consequences of growing in a hypoxic environment; these factors are the key to managing placental insufficiency. The prompt detection of fetuses compromised by IUGR and the determination of the degree of hemodynamic imbalance are crucial in preventing the greater risk of stillbirth, neonatal death and cerebral palsy seen in this population. Attention should therefore be given to developing new diagnostic and prognostic tools and efforts should be made to make such tools clinically useful. In this context, the study of AoI Doppler velocimetry is of particularly important relevance.

## Notes

**Erratum:** Author affliations have been corrected as per Erratum published on August 26, 2020. DOI of the Erratum is 10.1055/s-0040-1715592.
